# Diagnostic Performance of KLCA-NCC 2018 Criteria for Hepatocellular Carcinoma Using Magnetic Resonance Imaging: A Systematic Review and Meta-Analysis

**DOI:** 10.3390/diagnostics11101763

**Published:** 2021-09-25

**Authors:** Dong Hwan Kim, Bohyun Kim, Seo Yeon Youn, Hokun Kim, Joon-Il Choi

**Affiliations:** 1 Department of Radiology, Seoul Saint Mary’s Hospital, College of Medicine, The Catholic University of Korea, 222 Banpo-daero, Seocho-gu, Seoul 06591, Korea; kimdh@catholic.ac.kr (D.H.K.); baboojum@naver.com (B.K.); imseoyeon@gmail.com (S.Y.Y.); walehn@gmail.com (H.K.); 2 Cancer Research Institute, College of Medicine, The Catholic University of Korea, 222 Banpo-daero, Seocho-gu, Seoul 06591, Korea

**Keywords:** liver neoplasms, hepatocellular carcinoma, diagnosis, magnetic resonance imaging, meta-analysis

## Abstract

Several imaging-based systems have been proposed for the diagnosis of hepatocellular carcinoma (HCC) using magnetic resonance imaging (MRI), reflecting geographical differences in the clinical environment for HCC. We conducted a systematic review and meta-analysis to determine the performance of the Korean Liver Cancer Association-National Cancer Center (KLCA-NCC) 2018 criteria for the MRI diagnosis of HCC. Original studies reporting the performance of the KLCA-NCC 2018 criteria for the diagnosis of HCC using MRI were identified in MEDLINE and EMBASE until 29 March 2021. The meta-analytic pooled sensitivity and specificity of the KLCA-NCC 2018 criteria for diagnosing HCC were calculated using a bivariate random-effects model. A meta-regression analysis was performed to explore study heterogeneity further. Eight studies involving 1690 HCCs reported the accuracy of the KLCA-NCC 2018 imaging criteria. The pooled sensitivity and specificity of the definite HCC criteria for diagnosing HCC were 81% (95% confidence interval, 76–85%; *I*^2^ = 86%) and 90% (86–93%; *I*^2^ = 23%), respectively. For five available studies, the pooled sensitivity and specificity of the definite HCC criteria for diagnosing HCCs smaller than 20 mm were 80% (72–86%; *I*^2^ = 76%) and 91% (86–94%; *I*^2^ = 0%), respectively. A considerable threshold effect with a correlation coefficient of 0.667 was observed. The results of the meta-regression analysis revealed that the accuracy of the definite HCC criteria differed significantly depending on the type of MRI contrast agent (*p* = 0.01). In conclusion, the KLCA-NCC 2018 criteria had good overall diagnostic performance in diagnosing HCC. Substantial study heterogeneity was observed for sensitivity, which was significantly influenced by the type of contrast agent and by a threshold effect.

## 1. Introduction

Hepatocellular carcinoma (HCC) is the most common primary liver cancer and the third most frequent cause of cancer-related deaths [1,2]. HCC is the only malignancy that can be noninvasively diagnosed in at-risk patients by imaging, including multiphase computed tomography, contrast-enhanced ultrasonography, and magnetic resonance imaging (MRI), without pathological confirmation [3,4]. Therefore, accurate and consistent imaging-based diagnosis is of the utmost importance for the management of HCC. Worldwide, all imaging-based systems use a combination of arterial-phase hyperenhancement plus washout for the diagnosis of HCC [3,4,5]. However, geographical differences in the clinical environment and treatment practice make it challenging to establish universal guidelines for the diagnosis of HCC [3,4,5]. Unlike Western countries where liver transplantation is considered the only curative treatment, resection and loco-regional therapy are common curative options in Asia.

The Korean Liver Cancer Study Group (KLCSG)-National Cancer Center (NCC) HCC practice guidelines were developed in 2003 and updated in 2009, 2014, and 2018 [5,6,7,8]. Unlike prior versions, the Korean Liver Cancer Association (KLCA, formerly KLCSG)-NCC (KLCA-NCC) 2018 practice guidelines have been revised into a nonbinary system for the imaging diagnosis of HCC [5]. The KLCA-NCC 2018 guidelines categorize each hepatic lesion according to its likelihood of HCC, i.e., indeterminate nodules, probable HCC, and definite HCC [5]. A diagnosis of the definite HCC can be made when hepatic lesions ≥1 cm show arterial-phase hyperenhancement with delayed washout after the exclusion of benign lesions showing marked T2 hyperintensity or non-HCC malignancies showing a targetoid appearance [5]. In particular, washout is possible not only during the portal phase but also during the transitional phase and hepatobiliary phase (HBP) on MRI with a hepatobiliary contrast agent [5]. The KLCA-NCC 2018 criteria were intended to improve sensitivity in the detection of early-stage HCC, which is eligible for curative treatments such as surgical resection and local ablation.

Several studies, most of which were single-center studies, reported conflicting results regarding the performance of KLCA-NCC 2018 for the diagnosis of HCC [9,10,11,12,13]. Given the increased attention to and use of the KLCA-NCC 2018 criteria, it is time to assess clearly the performance of KLCA-NCC 2018 in the diagnosis of HCC and to understand the causes of heterogeneity between studies. Therefore, we conducted a systematic review and meta-analysis to assess the performance of the KLCA-NCC 2018 criteria for diagnosing HCC with MRI and to determine the factors associated with study heterogeneity.

## 2. Materials and Methods

This meta-analysis was performed in compliance with the Preferred Reporting Items for Systematic Reviews and Meta-Analyses guidelines [14] and was registered in PROSPERO (ID: CRD42021269626). The following literature search, study selection, data extraction, and study quality assessments were independently conducted by two reviewers (each having ≥10 years of experience in liver MRI), and any disagreements were resolved via consensus.

### 2.1. Literature Search Strategy

A literature search of the MEDLINE and EMBASE databases was conducted to identify studies investigating the diagnostic performance of the KLCA-NCC 2018 criteria for the dichotomous diagnosis of HCC based on dynamic contrast-enhanced MRI. The search queries included “Liver”, “Hepatocellular carcinoma”, “Korean Liver Cancer Association-National Cancer Center”, and “KLCA-NCC”. Supplementary Table S1 lists the search terms in detail. The beginning date for the search was set to 1 January 2018 to focus on original articles using the 2018 version of the KLCA-NCC. The literature search was updated until 29 March 2021. A manual evaluation of the searched articles was performed to narrow down the number of relevant articles. The search was limited to original studies on human subjects written in English. In order to expand the search, the bibliographies of articles were screened for other potentially eligible articles.

### 2.2. Inclusion and Exclusion Criteria

After duplicate articles were removed, the articles were reviewed regarding eligibility: (i) Population, patients at risk of HCC with focal hepatic lesions (≥1 cm); (ii) Index test, dynamic contrast-enhanced liver MRI; (iii) Reference standard, pathological diagnosis or imaging follow-up; (iv) Outcomes, sensitivity and specificity of definite HCC diagnosis according to the KLCA-NCC 2018 criteria; and (v) Study design, not limited. The exclusion criteria were as follows: (i) inclusion of a small number of patients (<10); (ii) animal studies, case reports, review articles, editorials, or scientific abstracts/conference proceedings; (iii) studies that were not within the field of interest of this study; and (iv) studies without sufficient details to construct a diagnostic 2-by-2 table of the imaging results and reference standard findings. Articles were first screened by titles and abstracts, and fully reviewed after the first screening.

### 2.3. Data Extraction and Quality Assessment

The following data were extracted: (i) study characteristics including authors, year of publication, and study design (prospective vs. retrospective); (ii) subject characteristics including sample size, age, sex, underlying liver disease, total number of hepatic lesions, and number of HCCs; (iii) MRI characteristics including indications for MRI, MRI scanner field strength, and type of contrast agent; (iv) image interpretation method (multiple reviewers with consensus vs. multiple independent reviewers); (v) reference standards for HCC and non-HCC; (vi) interobserver agreement (κ) for the categorization of hepatic lesions according to the KLCA-NCC 2018 criteria; and (vii) the accuracy of KLCA-NCC 2018 imaging criteria for the dichotomous diagnosis of HCC. The exact numbers of true positives, true negatives, false positives, and false negatives among hepatic lesions were extracted to determine the diagnostic accuracy of the criteria. If not distinctly mentioned, data were manually retrieved from tables and figures. If an article did not contain sufficient data, we contacted the corresponding authors by email to request additional information or clarification.

The Quality Assessment of Diagnostic Accuracy Studies (QUADAS-2) criteria [15] were used to assess the quality of the selected studies. The QUADAS-2 tool assesses study quality according to four different domains (patient selection, index test, reference standard, and flow and timing).

### 2.4. Data Synthesis

The meta-analytic pooled sensitivity and specificity were calculated using a bivariate random-effects and hierarchical summary receiver operating characteristic (HSROC) model. Heterogeneity between studies for sensitivity and specificity was assessed using the Higgins *I*^2^ statistic (*I*^2^ > 50%: substantial heterogeneity). The presence of a threshold effect was analyzed by visual assessment of the coupled forest plots of sensitivity and specificity as well as by calculating the Spearman correlation coefficient between the sensitivity and the false-positive rate (i.e., 1—specificity) [16]. A correlation coefficient > 0.6 was considered to represent a considerable threshold effect [16]. Deeks’ funnel plot and Deeks’ asymmetry test were used to assess the presence of publication bias.

A subgroup analysis was performed on studies reporting diagnostic performance for HCCs less than 20 mm in size and those reporting data on the “probable HCC” category in the KLCA-NCC 2018 imaging criteria. Additional subgroup analyses were performed for intra-individual comparative studies between the latest updated international guidelines. The pooled sensitivity and specificity of the KLCA-NCC 2018 criteria were compared to those of the Liver Imaging Reporting and Data System (LI-RADS) v2018, European Association for the Study of the Liver (EASL), and Asian Pacific Association for the Study of the Liver (APASL) guidelines [4,17,18] using joint-model bivariate meta-regression.

In order to investigate the causes of study heterogeneity further, a meta-regression analysis was performed. The following covariates were considered: (i) study design (prospective vs. retrospective); (ii) number of patients (<200 vs. ≥200); (iii) MRI scanner field strength (3.0 T only vs. 1.5 T or 3.0 T); (iv) MRI contrast agent (hepatobiliary contrast agent (HBA) only vs. extracellular contrast agent (ECA) or HBA); (v) image interpretation method (multiple independent reviewers vs. multiple reviewers with consensus); (vi) reference standard for HCC (pathology only vs. pathology or imaging follow-up); and (vii) reference standard for non-HCC (pathology only vs. pathology or imaging follow-up).

Using the κ and 95% confidence interval (CI) reported in the individual studies, the pooled κ with a 95% CI for the categorization of hepatic lesions according to the KLCA-NCC 2018 criteria was calculated using the DerSimonian–Laird random-effects model. κ was categorized according to the standards of Landis and Koch [19].

Stata version 16.0 (StataCorp LP, College Station, TX, USA) and R version 3.3.2 (The R Foundation for Statistical Computing, Vienna, Austria) were used for the analysis, with *p* < 0.05 considered statistically significant.

## 3. Results

### 3.1. Literature Search

**Figure 1:**
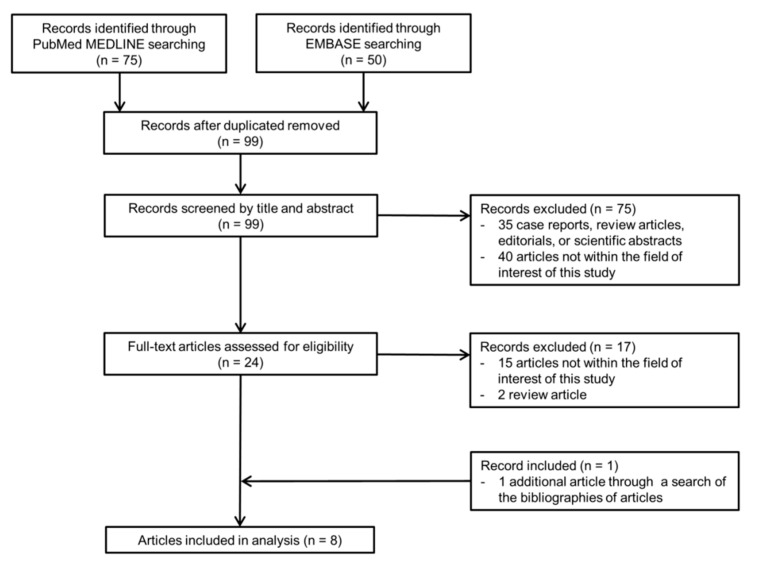
Flow diagram of the article selection process.

A total of 99 articles were screened after removing duplicates. Of these, 75 articles were excluded based on their titles and abstracts. Seventeen additional articles were excluded during a full-text review (Figure 1). A search of the bibliographies of the remaining articles yielded one additional eligible article. Ultimately, a total of eight eligible articles reporting the diagnostic performance of the KLCA-NCC 2018 criteria for the diagnosis of HCC were selected [9,10,11,12,13,20,21,22].

The characteristics of the final set of included articles are summarized in Table 1. Of the eight included articles, one study had a prospective design [22]. The most common underlying liver disease was hepatitis B in all eight included studies [9,10,11,12,13,20,21,22]. The indications for performing liver MRI were a pretransplant work-up in one study [10] and an evaluation of hepatic lesions detected during a surveillance examination in seven studies [9,11,12,13,20,21,22]. Two studies used only 3.0-T MRI scanners [11,20]. Six used only the HBA (gadoxetate disodium) [9,10,11,12,13,22], whereas two used both the HBA (gadoxetate disodium) and ECA (gadoterate meglumine, gadobutrol, or gadopentetate dimeglumine) [20,21]. Multiple image reviewers worked independently in five studies [10,11,12,13,22] and with consensus in three [9,20,21]. Six studies used pathology as the only reference standard for the diagnosis of HCC [10,11,12,13,20,21], whereas two used a combination of pathological diagnosis and imaging follow-up [9,22]. In all but one study [10], a combination of pathological diagnosis and imaging follow-up was used as a reference standard for non-HCC lesions.

### 3.2. Quality Assessment

The quality of the included articles is summarized in Supplementary Figure S1. In the flow and timing domain, one study had a high risk of bias due to an inappropriate interval between the index test and the reference standard (i.e., within 180 days) and did not consistently use the same reference standard [11]. In addition, three studies showed an unclear risk of bias because the interval between the index test and reference standard was not specified [12,13,22]. In the patient selection domain, one study showed a high risk of bias because it enrolled only patients who underwent liver transplantation [10]. In the reference standard domain, two studies left it unclear whether the reference standard results were interpreted without knowledge of the index test results [9,22].

### 3.3. Performance of the KLCA-NCC 2018 Criteria in Diagnosing HCC

**Figure 2:**
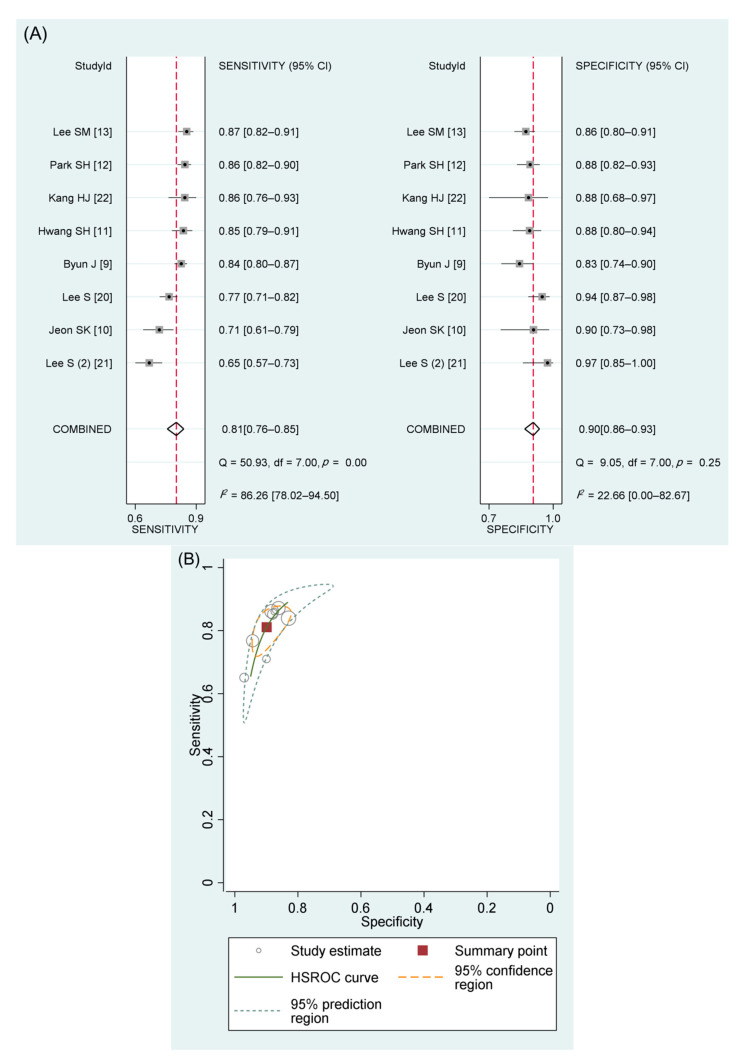
Coupled forest plots and HSROC curve for definite HCC of KLCA-NCC 2018 imaging criteria. (**A**) Coupled forest plots of the sensitivity and specificity of definite HCC for the diagnosis of HCC. (**B**) HSROC curve for the accuracy of definite HCC for the diagnosis of HCC. HSROC, hierarchical summary receiver operating characteristic; HCC, hepatocellular carcinoma; KLCA-NCC, Korean Liver Cancer Association-National Cancer Center; CI, confidence interval; df, degrees of freedom.

There were 1690 HCCs out of 2378 hepatic lesions in eight studies [9,10,11,12,13,20,21,22]. The meta-analytic summary sensitivity and specificity of definite HCC criteria were 81% (95% CI, 76–85%; *I*^2^, 86%) and 90% (95% CI, 86–93%; *I*^2^, 23%), respectively (Table 2 and Figure 2A). The HSROC curve with 95% confidence and prediction regions (Figure 2B) showed a large difference between the two regions, indicating considerable heterogeneity between studies. The area under the summary receiver operating characteristic curve was 0.93 (95% CI, 0.90–0.95). Visual analysis of the coupled forest plots of sensitivity and specificity indicated the presence of a threshold effect (Figure 2A), as did the corresponding correlation coefficient of 0.667 between the sensitivity and the false-positive rate (*p* = 0.07). There was no significant publication bias across the studies (*p* = 0.85; Supplementary Figure S2).

Five studies (478 HCCs out of 870 lesions) reported the performance of the definite HCC criteria in diagnosing HCCs less than 20 mm in size [9,10,12,13,20], showing a pooled sensitivity and specificity of 80% (95% CI, 72–86%; *I*^2^ = 76%) and 91% (95% CI, 86–94; *I*^2^ = 0%), respectively. Three studies (646 HCCs out of 957 lesions) reported the performance of the “probable HCC” category for diagnosing HCC [13,20,21]. The meta-analytic pooled sensitivity and specificity for a combination of the definite and probable HCC (definite/probable HCC) categories were 87% (95% CI, 81–92%) and 87% (95% CI, 81–93%), respectively.

### 3.4. Subgroup Analysis Comparing the Performance of Different International Guidelines

Six studies compared the diagnostic performance of the KLCA-NCC 2018 definite HCC criteria and the LI-RADS category 5 (LR-5) criteria [9,10,11,12,13,20]. The definite HCC criteria of KLCA-NCC 2018 demonstrated a significantly higher pooled sensitivity than the LR-5 criteria (82% (95% CI, 77–88%) vs. 65% (95% CI, 57–74%)) but a significantly lower pooled specificity (89% (95% CI, 85–92%) vs. 95% (95% CI, 92–97%)) (*p* = 0.01). Five studies compared the diagnostic performance of the KLCA-NCC 2018 definite HCC criteria and the EASL guideline [9,10,11,12,22]. A similar trend was observed, where the pooled sensitivity of the definite HCC criteria of KLCA-NCC 2018 was significantly higher than that of EASL guideline (83% (95% CI, 78–88%) vs. 60% (95% CI, 52–67%)), but the pooled specificity was significantly lower (87% (95% CI, 83–90%) vs. 93% (95% CI, 90–96%)) (*p* < 0.01). Four studies compared the diagnostic performance of the KLCA-NCC 2018 definite HCC criteria and the APASL guideline [9,10,11,12]. The definite HCC criteria of KLCA-NCC 2018 showed a significantly lower pooled sensitivity than the APASL guideline (83% (95% CI, 77–88%) vs. 87% (95% CI, 82–91%)) but a higher pooled specificity (87% (95% CI, 83–90%) vs. 80% (95% CI, 76–85%)) (*p* = 0.04).

### 3.5. Meta-Regression Analysis

The results of the meta-regression analysis for the diagnostic performance of KLCA-NCC 2018 are summarized in Table 3. Among the eight included covariates, the type of MRI contrast agent (*p* = 0.01) was the only factor that significantly influenced study heterogeneity. Studies using only HBA showed significantly higher sensitivity than those using either ECA or HBA (84% vs. 72%) but showed significantly lower specificity (87% vs. 95%). The other covariates were not significantly associated with study heterogeneity.

### 3.6. Interobserver Agreement for Categorization

Five included studies with a total of 1352 hepatic lesions (911 HCCs) reported the interobserver agreement for the categorization of lesions according to the KLCA-NCC 2018 criteria using MRI [10,11,13,20,21]. There was substantial or almost perfect agreement between image reviewers, with κ values ranging from 0.62 to 0.94. For available studies reporting the κ and standard variance (i.e., the CI of κ) [10,20,21], the pooled κ was 0.90 (95% CI, 0.84–0.96; *I*^2^ = 78%; Supplementary Figure S3).

## 4. Discussion

Our meta-analysis showed that the pooled sensitivity and specificity of the KLCA-NCC 2018 imaging criteria for definite HCC were 81% (95% CI, 76–85%) and 90% (95% CI, 86–93%), respectively. For HCCs smaller than 20 mm, the performance of these standards was comparable to its performance for all HCCs. For the definite/probable HCC categories, the meta-analytic summary sensitivity and specificity were 87% (95% CI, 81–92%) and 87% (95% CI, 81–93%), respectively. Substantial study heterogeneity was noted in sensitivity, with the type of MRI contrast agent and the threshold effect being significantly associated with study heterogeneity.

This study demonstrated that the definite HCC criteria of KLCA-NCC 2018 had good overall diagnostic performance for the MRI diagnosis of HCC, with substantial to almost perfect interobserver agreement. In addition, the subgroup analyses revealed that the pooled sensitivity of the definite HCC criteria of KLCA-NCC 2018 was significantly higher than that of the LR-5 or EASL guideline, consistent with recent comparative studies [9,10,11,12,13,20,22]. In line with previous studies [23,24], the high sensitivity of KLCA-NCC 2018 is largely attributable to the extended washout time, which extends to the transitional phase and the HBP. Since HBP signal intensity alterations precede typical vascular profile changes during hepatocellular carcinogenesis [25], more cases of early HCC can be diagnosed as definite HCC when an extended washout is used. The result that the APASL guideline had the highest pooled sensitivity may also be attributable to the application of HBP hypointensity as an alternative to the washout appearance. These higher sensitivities are particularly valuable in Asian countries, where HCC is highly prevalent, and surgical resection or image-guided ablation is preferred for HCC treatment [26,27]. However, the pooled specificity of the definite HCC criteria of KLCA-NCC 2018 was significantly lower than that of the LR-5 or EASL guideline. In the KLCA-NCC 2018 criteria, the specificity inevitably decreased as the sensitivity increased, which is a rather expected result stemming from the fundamental nature of diagnostic test accuracy. However, the pooled specificity of the KLCA-NCC 2018 guideline was significantly higher than that of the APASL guideline as a result of applying size criteria (i.e., ≥1 cm) and exclusion criteria for HCC, such as targetoid appearance, to exclude common causes of a false-positive diagnosis, including non-HCC malignancy. Consequently, unnecessary treatment or biopsy can be avoided through the application of the KLCA-NCC 2018 rather than the APASL guideline. Overall, we believe that the KLCA-NCC 2018 achieved an appropriate balance between sensitivity-oriented guideline such as the APASL guideline and specificity-oriented guidelines such as the LI-RADS or EASL guidelines and is more optimized for Asian countries than other international guidelines.

In our meta-regression analyses, the type of contrast agent was the only significant factor affecting study heterogeneity. The results revealed that the use of the HBA for MRI showed significantly better sensitivity for definite HCC than the mixed use of ECAs and HBAs, but it also showed lower specificity. The superior sensitivity of HBA-MRI may be attributable to the extended definition of washout, but this high sensitivity comes at the cost of specificity [28]. Although most Asian countries, to improve sensitivity for early detection of HCC, tend to use HBA-MRI more than ECA-MRI for the evaluation of HCC in clinical practice, consideration should be given for an improvement of the low sensitivity of ECA-MRI. As with LI-RADS, it can be optional to adopt additional major imaging features of definite HCC, such as an enhancing capsule and threshold growth. In addition, we noted a significant positive correlation between the sensitivity and the false-positive rate (correlation coefficient = 0.667). This result probably indicates that the study heterogeneity was partly due to the threshold effect that occurs when different thresholds or cutoff values are used to determine a positive test result [16]. For example, the included studies applied different washout criteria depending on the type of contrast agent used, and there might be some uncertainty about the definition of ancillary imaging features such as targetoid appearance.

This study has several limitations. First, the number of included studies was small (*n* = 8), as the KLCA-NCC 2018 criteria have only recently been introduced. Second, substantial study heterogeneity was noted regarding sensitivity, which could preclude the creation of robust meta-analytic estimates for diagnostic accuracy. To minimize this limitation, we investigated its causes and found that the type of contrast agent and the threshold effect significantly influenced study heterogeneity. Third, all included studies originated from South Korea, an area where hepatitis B is endemic, which limits the generalization of our results to other geographic populations with different etiologies of chronic liver disease. However, this geographic homogeneity brought somewhat homogeneous patient cohorts, which may enhance the quality of the evidence provided by this meta-analysis. Given that the KLCA-NCC criteria are focused on the Asian population in an area where hepatitis B is endemic, our meta-analysis represents the currently available evidence regarding its performance for the diagnosis of HCC.

## 5. Conclusions

In conclusion, the KLCA-NCC 2018 criteria had good overall diagnostic performance with balanced sensitivity and specificity in diagnosing HCC using MRI, even for HCCs smaller than 20 mm. In comparison with the definite HCC category alone, the definite/probable HCC category showed an increase in pooled sensitivity and a decrease in specificity. Substantial study heterogeneity was noted for sensitivity and was significantly associated with the threshold effect and the type of MRI contrast agent. In addition, studies that used HBAs showed significantly higher sensitivity but lower specificity than those that used ECAs to diagnose HCCs.

## Figures and Tables

**Table 1:** Characteristics of the included articles.

Author (Year of Publication)	Study Design	No. of Patients (% Male)	Patient Age, Years *	Most Common Etiology of Liver Disease (% of Cirrhosis)	No. of Hepatic Lesions	No. of HCC (%)	HCC Size, mm *	Indication for Liver MRI	MRI Magnet	MRI Contrast Agent (%)	Image Reviewer	Reference Standard for HCC	Reference Standard for Non-HCC
Byun J (2020) [9]	Retrospective	400 (80.5)	59.7	Hepatitis B virus (NA)	493	399 (80.9)	21 (range, 10–30)	Evaluation of hepatic nodule detected during surveillance	1.5- or 3.0-T	HBA	Multiple reviewers with consensus	Pathology or imaging follow-up	Pathology or imaging follow-up
Jeon SK (2020) [10]	Retrospective	81 (82.7)	54.1 ± 8.7	Hepatitis B virus (NA)	137	107 (78.1)	20 ± 16	Pretransplant work-up	1.5- or 3.0-T	HBA	Multiple independent reviewers	Pathology only	Pathology only
Lee S (2020) [20]	Retrospective	273 (68.9)	57.3 ± 9.5	Hepatitis B virus (54.9)	352	263 (74.7)	24 (range, 15–34)	Evaluation of hepatic nodule detected during surveillance	3.0-T	ECA or HBA	Multiple reviewers with consensus	Pathology only	Pathology or imaging follow-up
Lee S (2020) (2) [21]	Retrospective	142 (73.2)	57.2 ± 9.9	Hepatitis B virus (NA)	183	149 (81.4)	30.2 ± 23.9	Evaluation of hepatic nodule detected during surveillance	1.5- or 3.0-T	ECA or HBA	Multiple reviewers with consensus	Pathology only	Pathology or imaging follow-up
Hwang SH (2021) [11]	Retrospective	177 (80.2)	58	Hepatitis B virus (NA)	241	149 (61.8)	20, median	Evaluation of hepatic nodule detected during surveillance	3.0-T	HBA	Multiple independent reviewers	Pathology only	Pathology or imaging follow-up
Kang HJ (2021) [22]	Prospective	103 (78.6)	63.1	Hepatitis B virus (43.7)	103	79 (76.7)	28.2 (range, 11–114)	Evaluation of hepatic nodule detected during surveillance	1.5- or 3.0-T	HBA	Multiple independent reviewers	Pathology or imaging follow-up	Pathology or imaging follow-up
Lee SM (2021) [13]	Retrospective	387 (78.8)	59 ± 10	Hepatitis B virus (74.2)	422	234 (55.5)	32 ± 21 (all lesions)	Evaluation of hepatic nodule detected during surveillance	1.5- or 3.0-T	HBA	Multiple independent reviewers	Pathology only	Pathology or imaging follow-up
Park SH (2021) [12]	Retrospective	386 (76.2)	56.4 ± 10.3	Hepatitis B virus (70.2)	447	310 (69.4)	18.6 ± 6.3	Evaluation of hepatic nodule detected during surveillance	1.5- or 3.0-T	HBA	Multiple independent reviewers	Pathology only	Pathology or imaging follow-up

* Unless otherwise indicated, data are the mean ± standard deviation. HCC, hepatocellular carcinoma; MRI, magnetic resonance imaging; NA, not available; HBA, hepatobiliary contrast agent; ECA, extracellular contrast agent.

**Table 2:** Diagnostic performance of definite HCC criteria of KLCA-NCC 2018 in diagnosing HCC.

		For All HCCs						For HCCs Smaller than 20 mm					
Author	Total Number of Nodules	Number of Lesions				Sensitivity (95% CI)	Specificity (95% CI)	Number of Lesions				Sensitivity (95% CI)	Specificity (95% CI)
		*TP*	*FP*	*FN*	*TN*			*TP*	*FP*	*FN*	*TN*		
Byun J (2020) [9]	493	335	16	64	78	84% (80, 87)	83% (74, 90)	136	10	27	60	83% (77, 89)	86% (75, 93)
Jeon SK (2020) [10]	137	76	3	31	27	71% (61, 79)	90% (73, 98)	39	2	21	21	65% (52, 77)	91% (72, 99)
Lee S (2020) [20]	352	202	5	61	84	77% (71, 82)	94% (87, 98)	57	2	22	51	72% (61, 82)	96% (87, 100)
Lee S (2020) (2) [21]	183	97	1	52	33	65% (57, 73)	97% (85, 100)						
Hwang SH (2021) [11]	241	127	11	22	81	85% (79, 91)	88% (80, 94)						
Kang HJ (2021) [22]	103	68	3	11	21	86% (76, 93%)	88% (68, 97)						
Lee SM (2021) [13]	422	204	26	30	162	87% (82, 91)	86% (80, 91%)	40	14	5	114	89% (76, 96)	89% (82, 94)
Park SH (2021) [12]	447	267	16	43	121	86% (82, 90)	88% (82, 93%)	111	12	20	106	85% (77, 90)	90% (83, 95)
Higgins *I*^2^ for study heterogeneity						86%	23%					76%	0%
Meta-analytic summary estimate using the bivariate model						81% (76, 85)	90% (86, 93)					80% (72, 86) *	91% (86, 94) *

* The meta-analytic summary estimates were derived from five available studies. HCC, hepatocellular carcinoma; KLCA-NCC, Korean Liver Cancer Association-National Cancer Center; TP, true positive; FP, false positive; FN, false negative; TN, true negative; CI, confidence interval.

**Table 3:** Results of meta-regression analysis of the accuracy of definite HCC criteria of KLCA-NCC 2018 for diagnosing HCC.

		Meta-Analytic Summary Estimate		
Covariates	Subgroup	Sensitivity (95% CI)	Specificity (95% CI)	*p*-Value
Study design	Prospective (*n* = 1)	86% (74, 98)	88% (72, 100)	0.74
	Retrospective (*n* = 7)	80% (75, 86)	90% (86, 94)	
Number of patients	<200 (*n* = 4)	77% (70, 85)	91% (86, 96)	0.42
	≥200 (*n* = 4)	84% (79, 89)	88% (84, 93)	
MRI scanner field	3.0 T only (*n* = 2)	81% (72, 91)	91% (86, 97)	0.70
strength	1.5 T or 3.0 T (*n* = 6)	81% (75, 87)	89% (85, 93)	
MRI contrast agent	Hepatobiliary contrast agent (*n* = 6)	84% (80, 87)	87% (84, 90)	0.01
	Extracellular or hepatobiliary contrast agent (*n* = 2)	72% (63, 80)	95% (91, 99)	
Image interpretation	Multiple independent reviewers (*n* = 5)	84% (79, 89)	89% (84, 93)	0.27
method	Multiple reviewers with consensus (*n* = 3)	76% (69, 84)	92% (86, 97)	
Reference standard	Pathology only (*n* = 6)	80% (74, 86)	91% (87, 95)	0.25
for HCC	Pathology or imaging follow-up (*n* = 2)	85% (76, 93)	84% (74, 93)	
Reference standard	Pathology only (*n* = 1)	71% (54, 88)	90% (77, 100)	0.35
for non-HCC	Pathology or imaging follow-up (*n* = 7)	82% (78, 87)	90% (86, 94)	

HCC, hepatocellular carcinoma; KLCA-NCC, Korean Liver Cancer Association-National Cancer Center; CI, confidence interval; MRI, magnetic resonance imaging.

## Data Availability

All data accessed and analyzed in this study are available in the article and its Supplementary Materials.

## Supplementary Materials

The following are available online at https://www.mdpi.com/article/10.3390/diagnostics11101763/s1, Table S1: Search queries, Figure S1: Results of quality assessments of the articles according to the QUADAS-2 criteria, Figure S2: Deeks’ funnel plot to evaluate publication bias regarding definite HCC as defined by the KLCA-NCC 2018 imaging criteria, Figure S3: Forest plot of interobserver agreement for categorization of hepatic lesions according to the KLCA-NCC 2018 imaging criteria.
