# Supplementary material for: Diagnostic Performance of KLCA-NCC 2018 Criteria for Hepatocellular Carcinoma Using Magnetic Resonance Imaging: A Systematic Review and Meta-Analysis
Source: Diagnostics (Basel). 2021 Sep 25;11(10):1763. doi: 10.3390/diagnostics11101763 (PMC8534457; doi:10.3390/diagnostics11101763)

Supplementary Table S1. Search queries

No.	Search queries for MEDLINE
#1	("Liver Neoplasms"[Mesh] OR "Liver"[Mesh] OR "Carcinoma, Hepatocellular"[Mesh] OR "Hepatocellular carcinoma"[TW]) AND "Radiology Information Systems"[mesh]
#2	"KLCA-NCC"[TW] OR "Korean Liver Cancer Association-national Cancer Center"[TW] OR "Korean Liver Cancer Association-National Cancer Center"[TW] OR "KLCA-NCC 2018"[TW] OR "KLCA"[TW] OR "National Cancer Center" [TW]
#3	#1 OR #2
#4	Magnetic Resonanc*[TW] OR MRI[TW] OR MR[TW] OR CT[TW] OR Computed Tomography[TW]
#5	#3 AND #4
#6	#5 AND English[Lang] AND ("2018/01/01"[PDAT] : "3000/12/31"[PDAT])

No.	Search queries for EMBASE
#1	'Korean Liver Cancer Association-National Cancer Center '/exp
#2	'KLCA-NCC':ti,ab,kw,de OR 'Korean Liver Cancer Association-national Cancer Center':ti,ab,kw,de OR 'Korean Liver Cancer Association-National Cancer Center':ti,ab,kw,de OR 'KLCA-NCC 2018':ti,ab,kw,de OR 'KLCA':ti,ab,kw,de OR 'National Cancer Center':ti,ab,kw,de
#3	#1 OR #2
#4	('Magnetic Resonanc*' OR MRI OR MR OR CT OR 'Computed Tomography'):ab,ti,kw
#5	#3 AND #4
#6	#5 AND [english]/lim AND [2018-2021]/py

Supplementary Figure S1. Results of quality assessments of the articles according to the QUADAS-2 criteria

The methodological quality distribution of the articles is presented as the proportions of articles (0–100%) with a low (i.e., high quality), high, and unclear risk of bias and the proportions of articles with low (i.e., high quality), high, or unclear concerns regarding applicability for each domain.

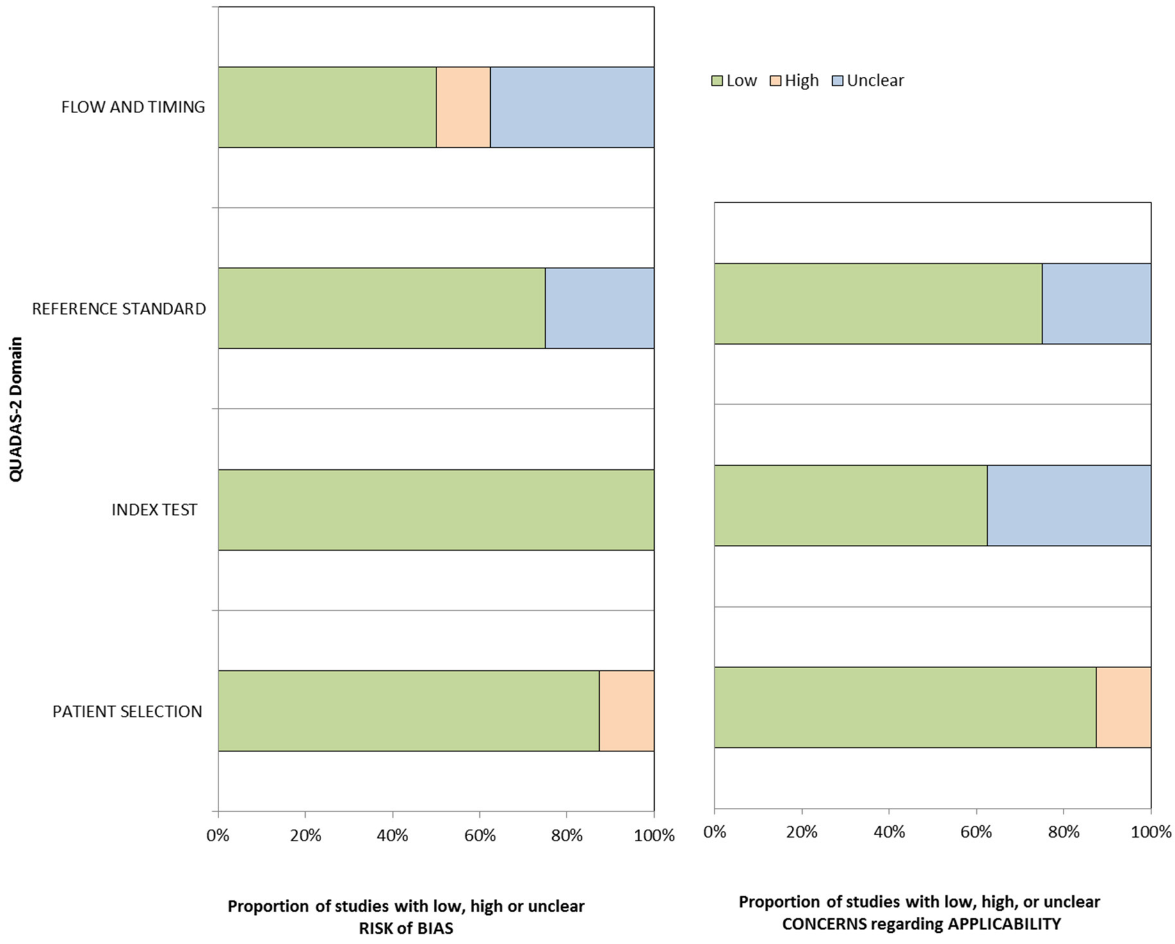

Supplementary Figure S2. Deeks' funnel plot to evaluate publication bias regarding definite HCC as defined by the KLCA-NCC 2018 imaging criteria

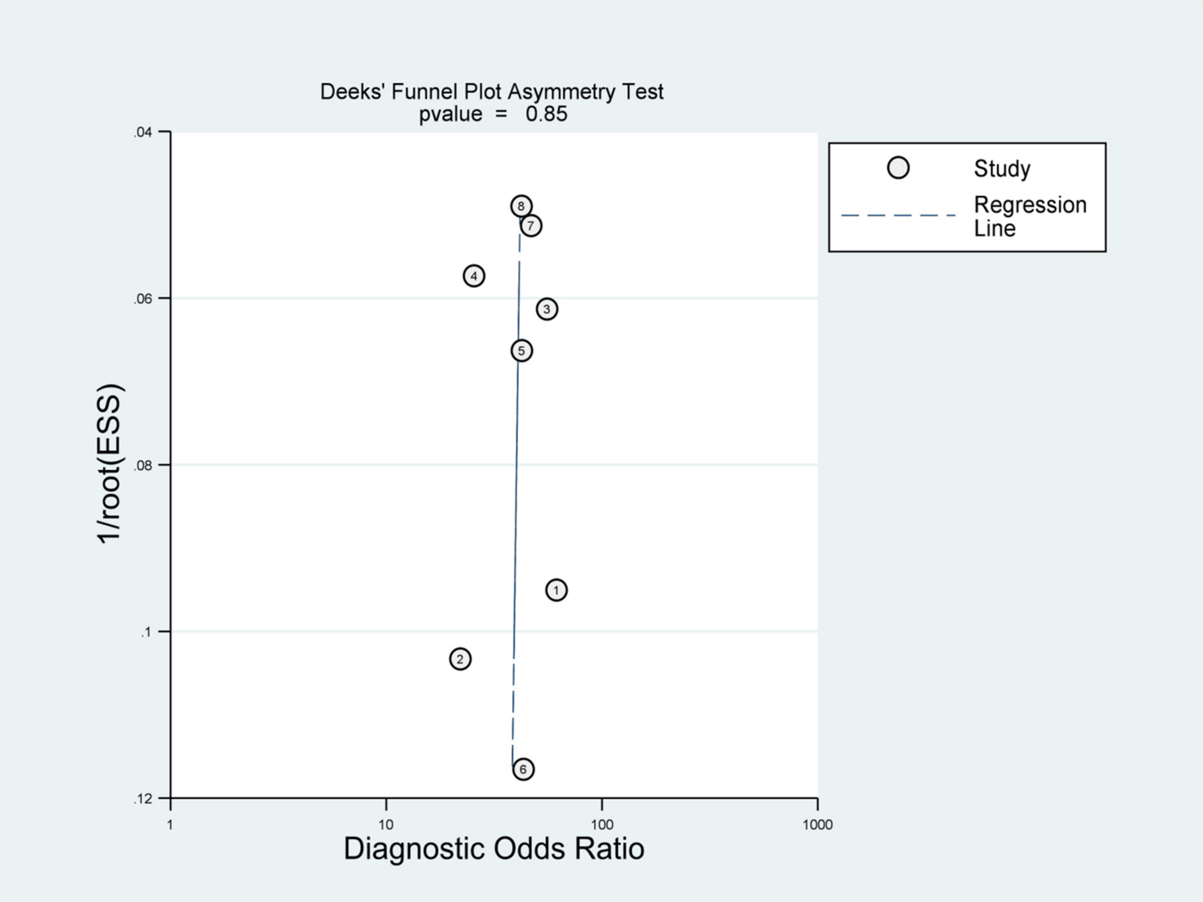

Supplementary Figure S3. Forest plot of interobserver agreement for categorization of hepatic lesions according to the KLCA-NCC 2018 imaging criteria.

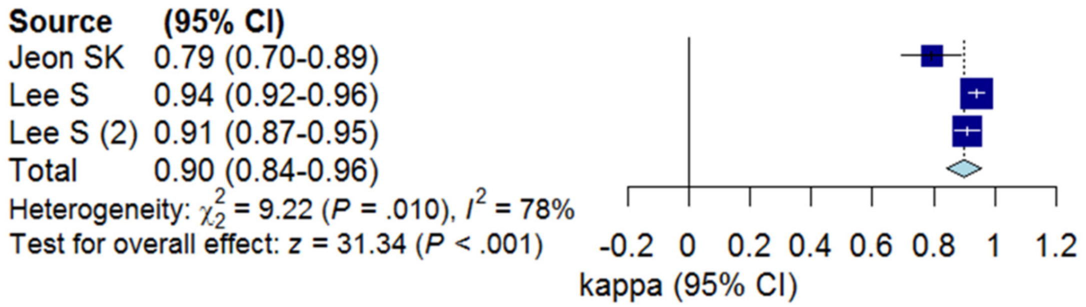

Supplement: Supplementary file 1 [file diagnostics-11-01763-s001.zip › diagnostics-1332003-SI.pdf]
